# Supplementary material for: Tudor-based proteomic strategy pan-specifically enriches and identifies protein arginine methylation
Source: EMBO Rep. 2025 Oct 20;26(23):5649–72. doi: 10.1038/s44319-025-00599-y (PMC12678802; doi:10.1038/s44319-025-00599-y)
Supplement: Supplementary file 1 — Appendix [file 44319_2025_599_MOESM1_ESM.pdf]

Appendix for

Tudor-based Proteomic Strategy Pan-specifically Enriches and Identifies Protein Arginine Methylation

Lingzi Lu#,[a], Ting Li#,[a], Rou Zhang#,[a], Yutong Wang[a] , Xiaoping Ye,[a] Yixin Luo,[a] Lingyu Sun[b], Liang Qi[a], Zilu Ye*[c], Yang Mao*[a], [d], Yanqiu Yuan*[a]

#These authors contributed equally

[a] L. Lu#, T. Li#, R. Zhang, X. Ye, Y. Wang, Y. Luo, Prof. Y. Mao, Prof. Y. Yuan
State Key Laboratory of Anti-Infective Drug Discovery and Development, School of Pharmaceutical Sciences, Sun Yat-sen University, Guangzhou, China
Email: maoyang3@mail.sysu.edu.cn (Y. Mao); yuanyq8@mail.sysu.edu.cn (Y. Yuan)

[b] L. Sun
Guangdong Institute for Drug Control, Guangzhou, China

[c] Prof. Z. Ye
State Key Laboratory of Common Mechanism Research for Major Diseases, Suzhou Institute of Systems Medicine, Chinese Academy of Medical Sciences & Peking Union Medical College, Suzhou, China
Email: yzl@ism.pumc.edu.cn

[d] Prof. Y. Mao
Guangdong Provincial Key Laboratory of Drug Non-Clinical Evaluation and Research, Guangzhou, China

Table of contents

Appendix Figure S1 ... 3
Appendix Figure S2 ... 4
Appendix Figure S3 ... 4
Appendix Figure S4 ... 4
Appendix Figure S5 ... 5
Appendix Figure S6 ... 5
Appendix Figure S7 ... 5

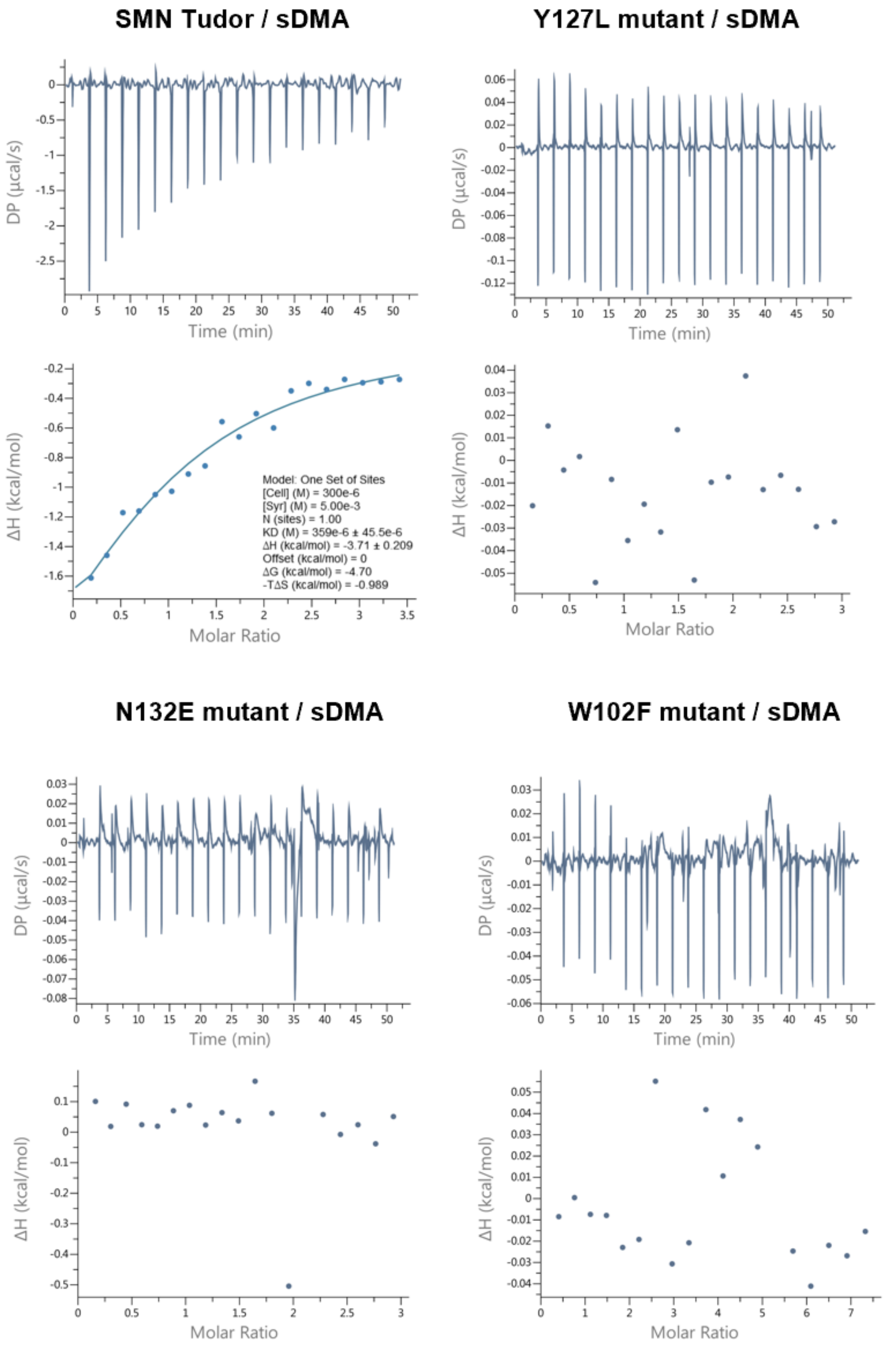

Appendix Figure S1. ITC titrations of wild type or mutant SMN Tudor with sDMA.

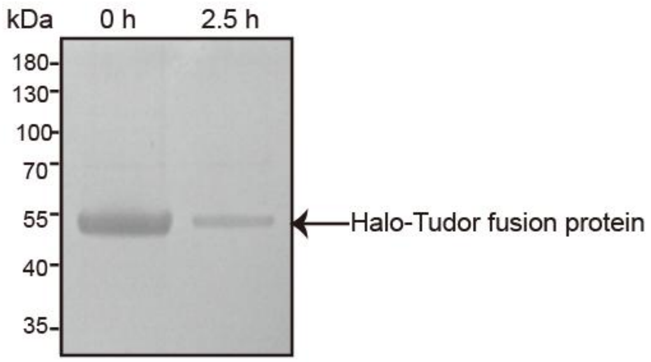

Appendix Figure S2. Coomassie blue staining after incubating 100 µL of Halo-link agarose resin with 360 µg of Halo-Tudor fusion protein at room temperature for 2.5 hours.

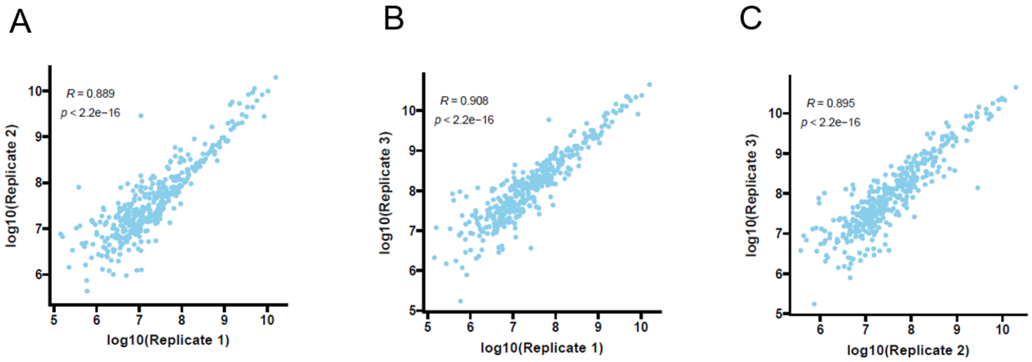

Appendix Figure S3. Correlation of the peptide precursor abundances. The Pearson correlation coefficient was used as a measure of linear correlation.

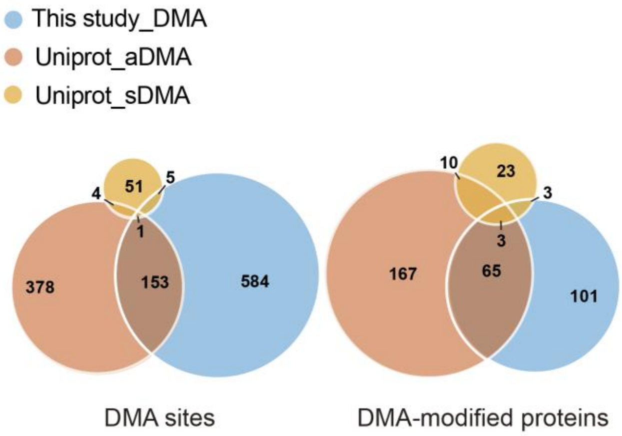

Appendix Figure S4. Venn diagram illustrates the overlapping sites or proteins containing DMA (both sDMA and aDMA) between our study and the UniProt database (2023_01).

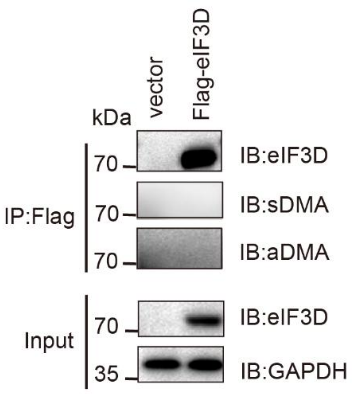

Appendix Figure S5. Immunoblot analysis of arginine dimethylation of immunoprecipitated Flag-eIF3D in HEK293T cells. Anti-sDMA and anti-aDMA antibodies were purchased from cell signaling technology with catalog numbers CST-13222 and CST-13522, respectively.

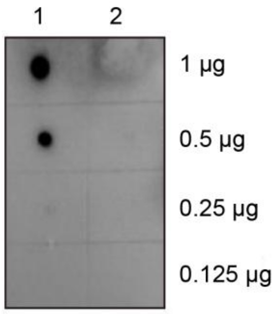

Appendix Figure S6. Dot blot analysis of anti-R99me2a antibody generated in the study. Lane 1: antigen Cys-RNRMR$_{me2a}$FAQRNL at different concentrations; Lane 2: antigen Cys-RNRMRFAQRNL at different concentrations. Antibody was diluted 1:4000.

A

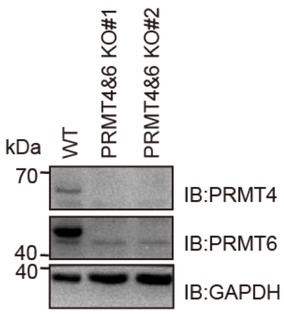

B

	PRMT4 genomic DNA	PRMT6 genomic DNA
Wild type	GGACATCATCATCTCGGAGCCCATGGGCTACATGCTCT	CGCCTGCGCGTGCTGCTGCGCTACAAAGTGGGAGACCA
PRMT4&6 KO#1	GGACATCATCATCTCGGAG----------------TCT (-16)	CGCCTGCGCGTGCTGCTGCGCTACAAAAGTGGGAGACCA (+1)
	GGACATCATCATCTCGGAGCC-ATGGGCTACATGCTCT (-1)	
PRMT4&6 KO#2	GGACATCATCATCTCGG----------CTACATGCTCT (-10)	CGCCTGCGCGTGCTGCTGCGCTAC------------CA (-12)
	GGACATCATCATCTCGGAGC--------TACATGCTCT (-8)	CGCCTGCGCGTGCTGCTGCGCTACAAAAGTGGGAGACCA (+1)

Appendix Figure S7. (A). Western blot analysis of PRMT4 and PRMT6 in both PRMT4 and PRMT6 double knockout HEK293T cell clones. GAPDH was used as a loading control. (B). Genomic DNA sequence of gRNA targeting site in both PRMT4 and PRMT6 double knockout HEK293T cell clones.
