# Supplementary material for: Tudor-based proteomic strategy pan-specifically enriches and identifies protein arginine methylation
Source: EMBO Rep. 2025 Oct 20;26(23):5649–72. doi: 10.1038/s44319-025-00599-y (PMC12678802; doi:10.1038/s44319-025-00599-y)
Supplement: Supplementary file 7 — Expanded View Figures [file 44319_2025_599_MOESM7_ESM.pdf]

Expanded View Figures

Figure EV1. ITC titrations of SMN Tudor protein with SmD1-derived heptapeptides or methylarginines.

The lines represent data fitting curves using 1:1 binding model at a fixed stoichiometry (N) of 1. The peptides used were SmD1 (AGRGRGR), SmD1-sDMA (AGR$_{me2s}$GRGR), SmD1 aDMA (AGR$_{me2a}$GRGR).

© The Author(s)

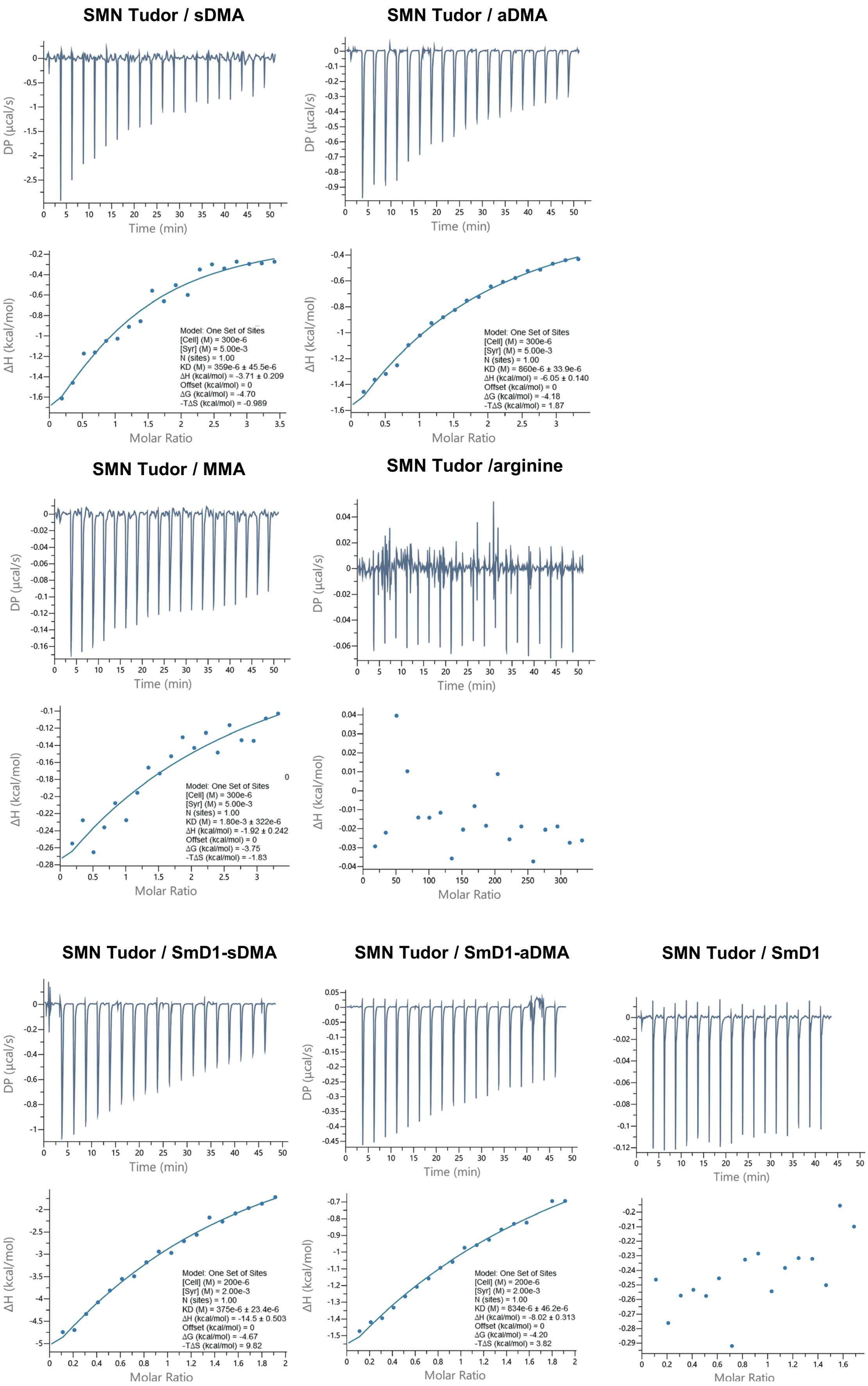
SMN Tudor / sDMA
DP (µcal/s)
Time (min)
ΔH (kcal/mol)
Molar Ratio
Model: One Set of Sites
[Cell] (M) = 300e-6
[Syr] (M) = 5.00e-3
N (sites) = 1.00
KD (M) = 359e-6 ± 45.5e-6
ΔH (kcal/mol) = -3.71 ± 0.209
Offset (kcal/mol) = 0
ΔG (kcal/mol) = -4.70
-TΔS (kcal/mol) = -0.989
SMN Tudor / aDMA
DP (µcal/s)
Time (min)
ΔH (kcal/mol)
Molar Ratio
Model: One Set of Sites
[Cell] (M) = 300e-6
[Syr] (M) = 5.00e-3
N (sites) = 1.00
KD (M) = 860e-6 ± 33.9e-6
ΔH (kcal/mol) = -6.05 ± 0.140
Offset (kcal/mol) = 0
ΔG (kcal/mol) = -4.18
-TΔS (kcal/mol) = 1.87
SMN Tudor / MMA
DP (µcal/s)
Time (min)
ΔH (kcal/mol)
Molar Ratio
Model: One Set of Sites
[Cell] (M) = 300e-6
[Syr] (M) = 5.00e-3
N (sites) = 1.00
KD (M) = 1.80e-3 ± 322e-6
ΔH (kcal/mol) = -1.92 ± 0.242
Offset (kcal/mol) = 0
ΔG (kcal/mol) = -3.75
-TΔS (kcal/mol) = -1.83
SMN Tudor /arginine
DP (µcal/s)
Time (min)
ΔH (kcal/mol)
Molar Ratio
SMN Tudor / SmD1-sDMA
DP (µcal/s)
Time (min)
ΔH (kcal/mol)
Molar Ratio
Model: One Set of Sites
[Cell] (M) = 200e-6
[Syr] (M) = 2.00e-3
N (sites) = 1.00
KD (M) = 375e-6 ± 23.4e-6
ΔH (kcal/mol) = -14.5 ± 0.503
Offset (kcal/mol) = 0
ΔG (kcal/mol) = -4.67
-TΔS (kcal/mol) = 9.82
SMN Tudor / SmD1-aDMA
DP (µcal/s)
Time (min)
ΔH (kcal/mol)
Molar Ratio
Model: One Set of Sites
[Cell] (M) = 200e-6
[Syr] (M) = 2.00e-3
N (sites) = 1.00
KD (M) = 834e-6 ± 46.2e-6
ΔH (kcal/mol) = -8.02 ± 0.313
Offset (kcal/mol) = 0
ΔG (kcal/mol) = -4.20
-TΔS (kcal/mol) = 3.82
SMN Tudor / SmD1
Time (min)
Molar Ratio

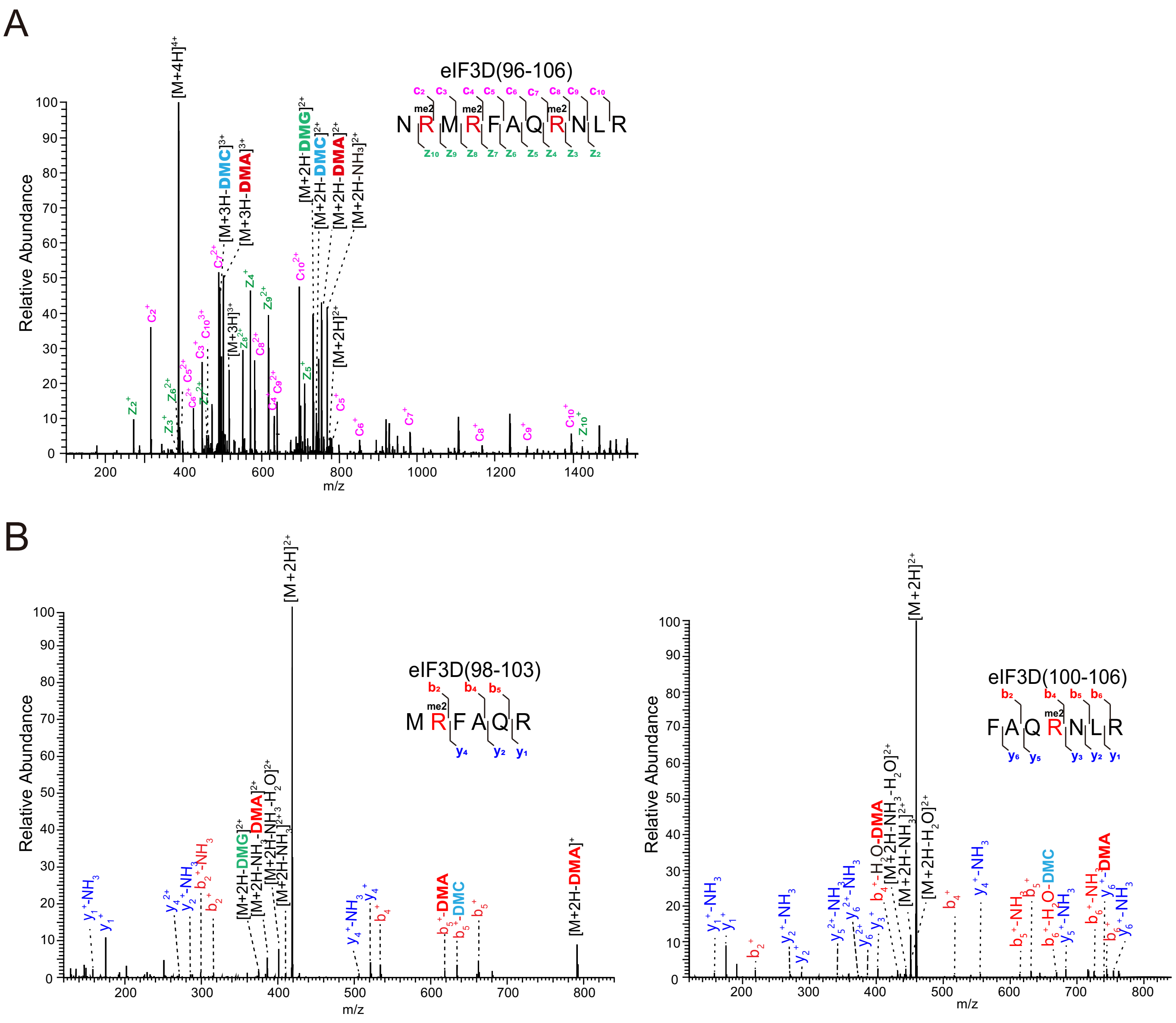

Figure EV2. MS/MS spectrums of the dimethylated peptide from eIF3D.

(**A**) MS/MS spectrum of the eIF3D peptide NR$_{me2}$MR$_{me2}$FAQR$_{me2}$NLR (96–106) identified by our molecular affinity approach. The spectrum was manually annotated with potential neutral losses. DMA, dimethylamine; DMC, dimethylcarbodiimide; DMG, dimethylguanidine. The abbreviations include their corresponding ions. (**B**) MS/MS spectrums of the eIF3D peptide MR$_{me2}$FAQR (98–103) and FAQR$_{me2}$NLR (100–106) from eIF3D by IP-MS. Plasmid constructs encoding the Full-length Flag-eIF3D proteins were transfected into HEK293T cells. eIF3D was immunoprecipitated with Flag agarose beads, digested with trypsin and subsequently subjected to mass spectrometry analysis.

 © The Author(s)

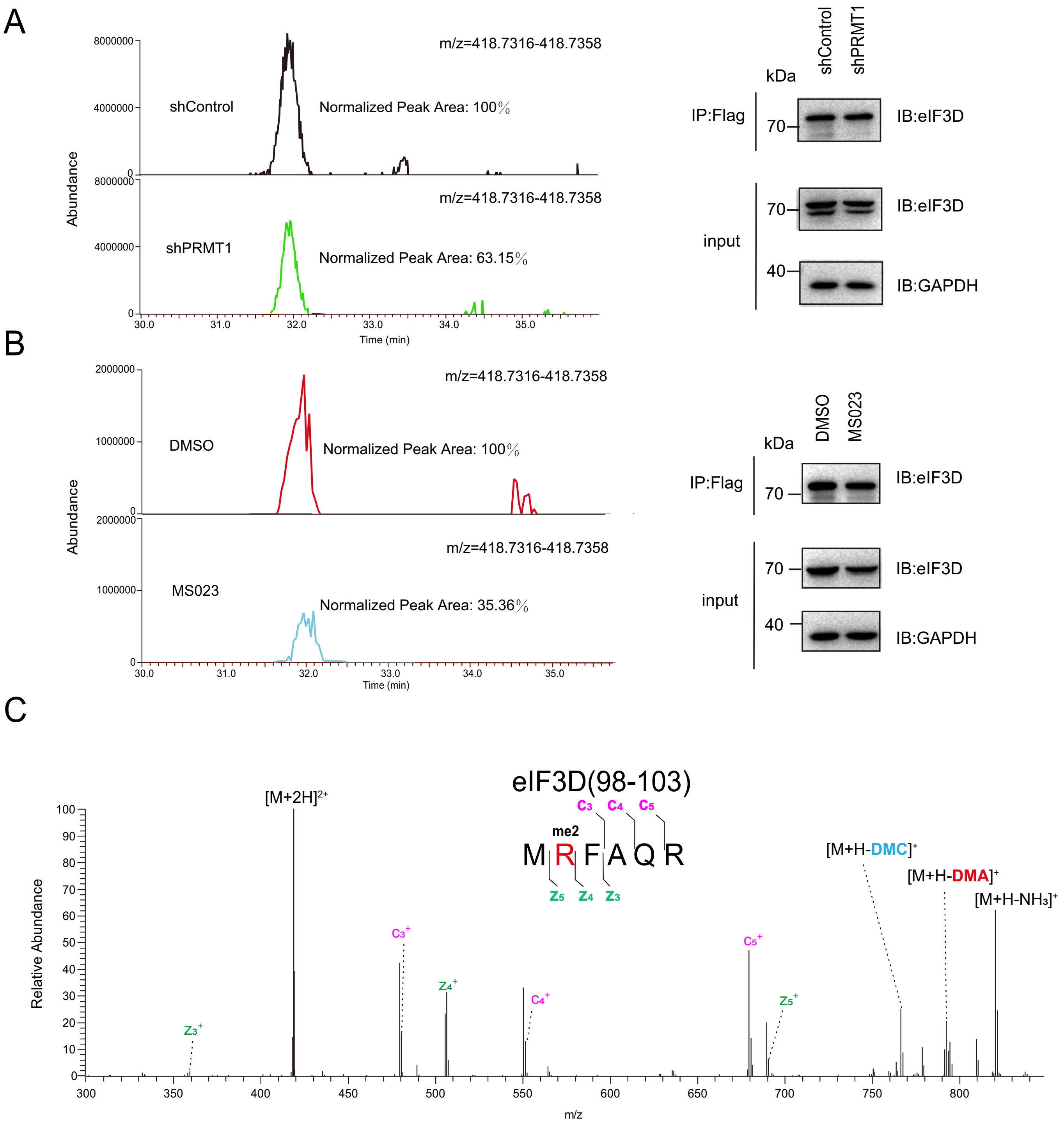

Figure EV3. IP-MS analysis of eIF3D R99 dimethylation in HEK293T cells.

(**A**) Quantitative MS analysis of eIF3D peptide ($MR_{me2}FAQR$) with and without PRMT1 knockdown. (**B**) Quantitative MS analysis of eIF3D peptide ($MR_{me2}FAQR$) with pharmacological inhibition of Type I PRMTs. Inhibitor treatment was performed with HEK293T cells overexpressing wild-type eIF3D with 1 μM MS023 for 48 h. (**C**) MS/MS spectrum of eIF3D peptide ($MR_{me2}FAQR$) from the IP-MS experiment. The spectrum was manually annotated with potential neutral losses. DMA, dimethylamine; DMC, dimethylcarbodiimide. The abbreviations include their corresponding ions.

© The Author(s)

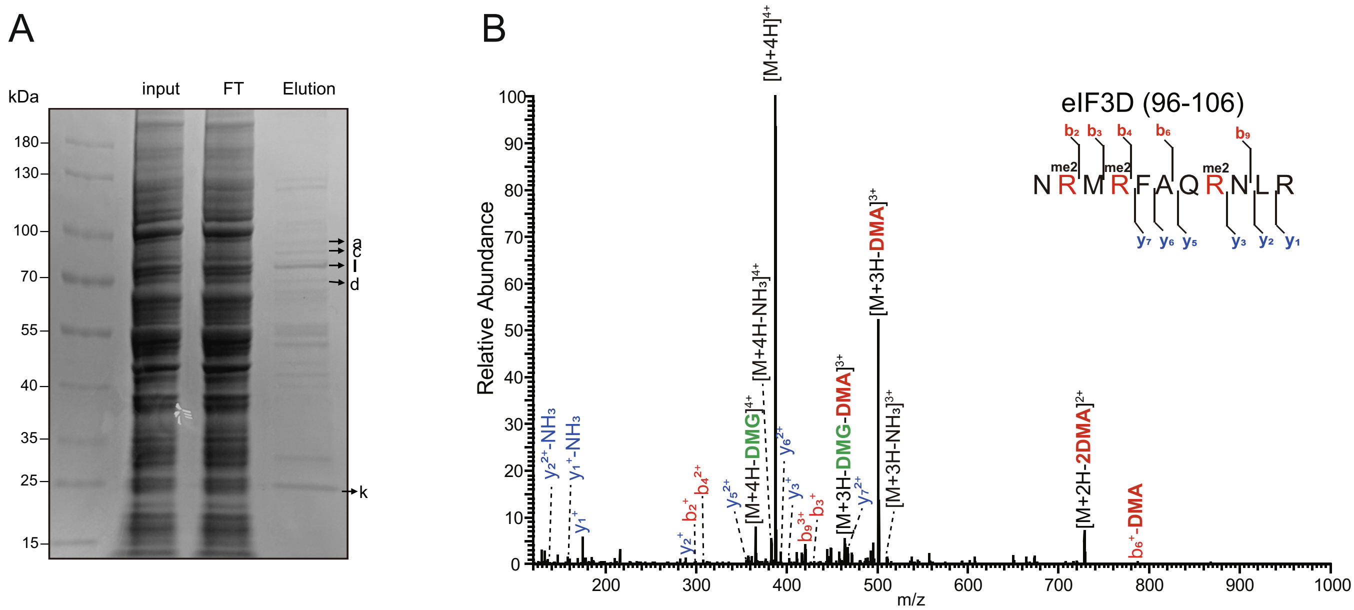

Figure EV4. Analysis of eIF3D arginine methylation from eIF3 complex.

(**A**) Coomassie stained SDS-PAGE gel showing purified eIF3 complex. (**B**) MS/MS spectrums of dimethylated eIF3D peptides $NR_{me2}MR_{me2}FAQR_{me2}NLR$ (96–106) from immunoprecipitated eIF3 complex. Plasmid constructs encoding the Full-length Flag-Myc-eIF3L proteins were transfected into HEK293T cells. eIF3L was immunoprecipitated with Flag agarose beads. The immunoprecipitated protein was divided into two aliquots: one portion was analyzed by Coomassie stain, and the other portion was digested with trypsin and subjected to mass spectrometry analysis. The spectrum was manually annotated with potential neutral losses. DMA, dimethylamine; DMG, dimethylguanidine. The abbreviations include their corresponding ions.

 © The Author(s)
